# Alpha-1 antitrypsin–glucocorticoid receptor axis: a new pathway in immune modulation

**DOI:** 10.1186/s10020-026-01470-z

**Published:** 2026-04-01

**Authors:** Kevin Möhlis, Julia Held, Elena Korenbaum, Sabine Wrenger, Sabina Janciauskiene, John T. Heiker

**Affiliations:** 1 Helmholtz Institute for Metabolic, Obesity and Vascular Research (HI-MAG) of the Helmholtz Zentrum München at the University of Leipzig, University Hospital Leipzig, Philipp-Rosenthal-Straße 27, Leipzig, 04103 Germany; 2 Department of Respiratory Medicine, Hannover Medical School, Feodor- Lynen-Str. 23, Hannover, 30625 Germany; 3 Biomedical Research in Endstage and Obstructive Lung Disease Hannover (BREATH), German Center for Lung Research (DZL), Hannover, Germany; 4 Division of Structural Biochemistry, Hannover Medical School, Carl- Neuberg-Str. 1, Hannover, 30625 Germany; 5 Institute of Biochemistry, Faculty of Life Sciences, University of Leipzig, Talstraße 33, Leipzig, 04103 Germany

**Keywords:** Alpha1-antitrypsin, Glucocorticoid receptor, Macrophages, PBMCs, Confocal microscopy, Immunoprecipitation

## Abstract

**Background:**

Alpha-1 antitrypsin (AAT) has been reported to interact with the glucocorticoid receptor (GR) and modulate its signaling. We extended these findings by testing whether native (nAAT) and oxidized AAT (oxAAT) bind GR.

**Methods:**

Binding of native and modified AAT (oxidized and cleaved) to GR and candidate receptors (LRP1, SR-B1, TfR, CD36) was assessed by ELISA. AAT-GR colocalization was examined in PBMCs and macrophage models by confocal microscopy and co-immunoprecipitation. *NR3C1* (GR) mRNA in PBMCs was analyzed after 24 h of treatment with AAT or dexamethasone.

**Results:**

nAAT and oxAAT bound GR (EC₅₀ 0.9 and 2.6 µM) and LRP1 (EC₅₀ 2.9 and 1.3 µM), whereas binding to SR-B1, TfR, and CD36 was weak (EC₅₀ > 5 µM). Cleaved AAT showed no binding. AAT-GR colocalization was present in non-activated PBMCs but absent in macrophages, where GR was predominantly nuclear. Both nAAT and oxAAT significantly reduced *NR3C1* mRNA, similar to dexamethasone.

**Conclusion:**

nAAT and oxAAT, but not cleaved forms of AAT, bind GR in vitro and associate with cytoplasmic GR in non-activated immune cells. Our results support the role of AAT in regulating GR signaling and highlight the AAT-GR axis as a putative mechanism of immune regulation.

**Supplementary Information:**

The online version contains supplementary material available at 10.1186/s10020-026-01470-z.

## Background

The glucocorticoid receptor (GR, encoded by the gene *NR3C1* (Nuclear Receptor Subfamily 3 Group C Member 1) is a ubiquitously expressed nuclear receptor and transcription factor present in most vertebrate cells. It mediates cellular responses to glucocorticoids and regulates a broad range of physiological processes, including metabolism, stress responses, and immune functions (Caratti et al. 2015). In resting cells, GR shows distinct distributions between the cytoplasm and the nucleus (Nicolaides et al. 2010). Upon binding glucocorticoid hormones (e.g., cortisol, dexamethasone), GR undergoes a conformational change and dissociates from its cytoplasmic chaperone complex (Heat Shock Protein 90 (HSP90), Heat Shock Protein 70 (HSP70), and FK506 Binding Protein 5/4 (FKBP51/52), allowing receptor dimerization and nuclear translocation. Once in the nucleus, GR regulates gene expression either by directly binding to glucocorticoid response elements in the DNA or indirectly through interactions with other transcription factors (Vockley et al. 2016).

GR represses pro-inflammatory gene expression (e.g., tumor necrosis factor alpha (*TNFA*), interleukin 1 beta (*IL1B*)) partly by antagonizing nuclear factor kappa-light-chain-enhancer of activated B cells (*NF-κB*) and activator protein 1 (AP-1), while inducing anti-inflammatory mediators such as interleukin 10 (*IL10*) (Iyer and Cheng 2012). However, because NF-κB pathway can also regulate anti-inflammatory genes, the functional outcome of GR-NF-κB interaction depends on the cellular context, including cell type, activating stimulus, and the availability of transcriptional co-regulators (De Bosscher et al. 2003). Beyond inflammation, GR regulates genes controlling cell cycle progression, differentiation, and glucose metabolism, underscoring its central role in coordinating immune and metabolic responses (Caratti et al. 2015; Kadmiel and Cidlowski 2013).

In addition to classical steroid ligands, GR can bind non-steroidal compounds that act as selective GR modulators with agonistic, partial agonistic, antagonistic, or dissociated transactivation/transrepression properties. Small molecules such as MK-593 and benzimidazole derivatives have been developed to selectively modulate GR signaling (Sundahl et al. 2015). These non-steroidal ligands provide a strategy to expand GR-targeted therapies while overcoming limitations of conventional glucocorticoids (Clarisse et al. 2024).

A recent study by Bai et al. was the first to show that human alpha-1 antitrypsin (AAT; *SERPINA1*), an acute phase glycoprotein, binds the GR in vitro and in macrophage models (Bai et al. 2022), and that AAT regulates GR-dependent genes involved in host immunity (Bai et al. 2024). Traditionally, AAT is recognized as a major inhibitor of neutrophil elastase and proteinase 3, but it also inhibits caspase-3, a central mediator of apoptosis, and ADAM17, a disintegrin and metalloproteinase 17, also known as TACE (Tumor necrosis factor-α-converting enzyme), which regulates the shedding of membrane-bound growth factors and cytokines, including TNFα (Janciauskiene et al. 2011; Lockett et al. 2013). Given that AAT directly modulates GR signaling, its inherited deficiency may contribute both to protease imbalance (Tuder et al. 2010) and reduced glucocorticoid responsiveness. In fact, AAT is shown to exert broad anti-inflammatory, antioxidant, and immunomodulatory effects, supporting inflammation resolution independently of protease inhibition (Farber et al. 2025; Jonigk et al. 2013; Ehlers 2014). Hence, the AAT-GR interaction may help to explain the broad anti-inflammatory effects of AAT observed in vitro and in vivo (Janciauskiene et al. 2018; Oshins et al. 2025).

Immune and structural cells can contain intracellular AAT either through endogenous *SERPINA1* expression or via uptake of circulating AAT by receptor-mediated endocytosis. Several uptake pathways have been described, including those mediated by low-density lipoprotein receptor-related protein 1 (LRP1), clathrin-coated vesicles, caveolae, and scavenger receptor class B type I (SR-BI) (Sohrab et al. 2009; Lockett et al. 2015; Serban and Petrache 2016). Once internalized, AAT can modulate intracellular signaling, for example by stabilizing NF-κB inhibitor alpha (IKBA) and limiting NF-κB activation (Zhou et al. 2011). Hence, AAT interaction with GR and influence on NF-κB (Bai et al. 2022) suggests that the AAT-GR axis may be important for the anti-inflammatory and immunomodulatory effects of AAT.

AAT contains exposed methionine (Met) residues, making it highly susceptible to oxidation (Levine et al. 1996). Oxidation of specific residues, particularly Met351 and Met358, impairs AAT’s anti-protease activity (Taggart et al. 2000; Beatty et al. 1980) and can alter its immunomodulatory properties (Mazzuca et al. 2024). OxAAT has been detected in patients with chronic obstructive pulmonary disease (COPD), cystic fibrosis, asthma, sepsis, and rheumatoid arthritis (Li et al. 2009; Topic et al. 2018), and has been proposed as a biomarker for oxidative stress. As a matter of fact, some experimental studies showed that oxAAT can exert anti-inflammatory effects similar to nAAT (Mazzuca et al. 2024; Zemtsovski et al. 2024), highlighting the complexity of AAT biology.

Given that both, nAAT and oxAAT, are present in vivo and exhibit overlapping biological activities (Sohrab et al. 2009; Lockett et al. 2014; Lewis 2012), we hypothesized that both forms may interact with GR. Building on the findings by Bai and co-authors (Bai et al. 2022), we tested whether native, oxidized, and cleaved AAT bind GR in vitro and compared this with other proposed AAT-binding receptors. We also investigated AAT-GR interaction in monocyte-derived macrophages and human peripheral blood mononuclear cells (PBMCs) and assessed whether AAT regulates GR (*NR3C1*) expression.

## Materials and methods

### Preparations of nAAT and oxAAT

**Fig. 1:**
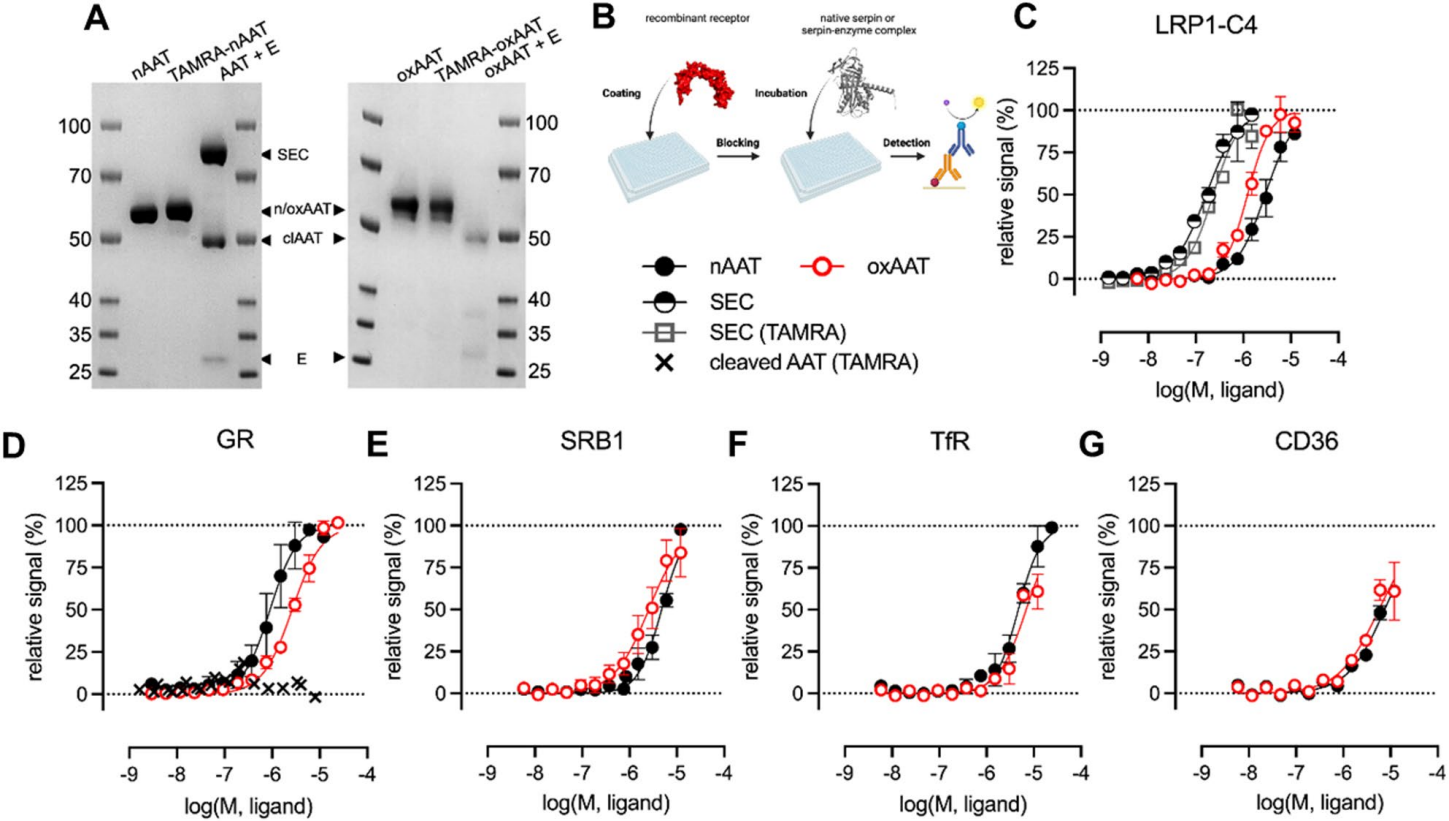
ELISA-based binding studies of native, oxidized, cleaved and complexed AAT. Candidate receptors LRP1, SR-B1, TfR, and CD36 were selected based on previous reports of AAT binding or uptake. This approach allowed us to compare AAT binding to GR with other proposed receptor pathways and assess specificity. **A** SDS-PAGE gel showing native (nAAT) and oxidized (oxAAT), TAMRA-labeled, complexed (AAT-elastase, so called serpin enzyme complex, SEC) and cleaved AAT (nAAT/oxAAT + elastase) preparations. Bands of the SEC, native/oxidized AAT (nAAT/oxAAT), cleaved AAT (clAAT) and elastase (E) are indicated. **B** ELISA set up to analyze AAT (native, oxidized, SEC (AAT-elastase), or cleaved AAT) binding to LRP1-C4 (cluster IV) (**C**), the GR (**D**), SR-B1 (**E**), TfR (**F**), and CD36 (**G**). Data are presented as mean ± SEM from at least two independent experiments, each with a minimum of four technical replicates, and were analyzed using nonlinear regression. Panel B was created in BioRender, https://BioRender.com/9oxpr9u

The nAAT was prepared from the therapeutic preparation Respreeza (CSL Behring, Marburg, Germany) after buffer-exchange into sterile phosphate-buffered saline (PBS, Thermo Fisher Scientific, Carlsbad, CA, USA) using Vivaspin-20 centrifugal filter units (10 kDa molecular weight cutoff; Sartorius, Göttingen, Germany). OxAAT was generated by incubating Respreeza-derived AAT with N-chlorosuccinimide (NCS, Sigma-Aldrich, Merck, Darmstadt, Germany) at a molar ratio of 1:20 (AAT: NCS) for 30 min at room temperature. Following oxidation, samples were washed four times with PBS using Vivaspin-20 centrifugal filters (10 kDa cutoff) to remove residual NCS. Protein concentrations were measured using the BCA Protein Assay (Thermo Fisher Scientific) according to the manufacturer’s instructions. OxAAT does not inhibit elastase activity (Moraga and Janciauskiene 2000) and accordingly did not form complex with elastase (Sigma Aldrich Chemie GmbH, Steinheim, Germany, #E7885, specific activity ≥ 4 units/mg protein, Fig. 1A).

### Generation of labeled and unlabeled elastase-complexed and cleaved AAT

AAT-elastase complex was generated by incubating AAT with porcine pancreas elastase (Sigma-Aldrich, Merck) at a molar ratio of 1:2 (AAT: elastase) for 30 min at room temperature. Elastase cleavage of AAT generates a covalent AAT-elastase complex (serpin-enzyme complex - SEC) (Fig. 1A). Conformational changes in AAT stabilize the complex and prevent release of the N-terminal cleaved fragment from elastase (Elliott et al. 1996). Notably, β-mercaptoethanol does not disrupt the complex because it is maintained by a covalent serpin-protease linkage (Lawrence et al. 1995). Cleaved AAT (clAAT) was generated by incubating oxAAT, which lacks inhibitory activity (Janciauskiene et al. 2018) (Fig. 1A), with elastase at a higher molar ratio (10:1 AAT: elastase). Under these conditions, elastase cleaves AAT without forming the SEC. No protease inhibitors were included, and elastase activity was not directly measured. While a potential interference of residual elastase activity cannot be entirely excluded, based on our prior experience with the LRP1 assays (Tindall et al. 2024) and the consistent detection of LRP1-SEC interaction in the present study, we consider such interference unlikely under the applied experimental conditions.

For some GR binding experiments, the synthetic C-terminal fragment of AAT (36 amino acid, C36 peptide > 95% purity, sb-Peptide, Saint Egrève, France) was used, which is a copy of C36 generated during AAT-elastase complex formation when AAT is cleaved at the reactive center loop generating large N-terminal and short C-terminal fragments of AAT. Moreover, in some experiments AAT was fluorescently labeled prior to complex formation or cleavage. Labeling was performed at Cys232 using tetramethylrhodamine-5(6)-C2-maleimide (TAMRA; AnaSpec, Fremont, CA, USA), as described previously (Tindall et al. 2024).

### ELISA-based AAT-receptor binding assay

The binding affinities of human nAAT and modified forms of AAT, including oxAAT, AAT-elastase-complexed and cleaved, toward various receptors were determined using an ELISA-based approach (Fig. 1B), as previously described (Tindall et al. 2024; Rapöhn et al. 2025). Recombinant proteins used included human LRP1 cluster IV (#5395-L4), cluster of differentiation 36 (CD36; #1955-CD), SR-BI (#8114-SRB), and transferrin receptor (TfR, #2474-TR) all from R&D Systems, Minneapolis, MN, USA), and human glucocorticoid receptor (GR; #A15663, Thermo Fisher Scientific), as previously described (Bai et al. 2022). All recombinant receptor proteins were reported to be suitable for ligand-binding studies according to the manufacturer or published data. Briefly, 384-well high-binding clear-bottom plates (#781061, Greiner, Kremsmünster, Austria) were coated with recombinant receptor proteins (500 ng/mL) in Tris-buffered saline (TBS) containing 5 mM CaCl₂, pH 8.0, to ensure consistent surface immobilization across wells.

After coating, the wells were blocked with 5% nonfat dry milk in TBS for 2 h at room temperature. Increasing concentrations of proteins were added to individual wells and incubated for 2 h at room temperature. Protein detection was performed by incubation with a primary anti-AAT antibody (Dako, Agilent, Santa Clara, CA, USA) or an anti-tetramethylrhodamine (TRITC) antibody (Invitrogen, Rockford, IL, USA) for 1.5 h, followed by incubation with a HRP-conjugated secondary rabbit antibody (Cell Signaling, Danvers, MA, USA) for 1 h. Signal development was achieved by addition of the HRP substrate 3,3′,5,5′-tetramethylbenzidine (TMB; Merck). The reaction was stopped with 0.16 M H_2_SO_4,_ and the absorbance was measured at 450 nm using a Flexstation 3 multimode microplate reader (Molecular Devices, San Jose, CA, USA). Each interaction was assessed using a minimum of four technical replicates within each of at least two independent binding assays (biological replicates). Technical replicates refer to multiple measurements of the same sample in different wells of the same plate within a single experiment to account for variability in the assay procedure, such as pipetting or plate handling. Absorbance values were plotted against log (molar concentration), and the half maximal effective concentration (EC₅₀) values were calculated by nonlinear regression analysis (log[agonist] vs. response) using Prism 10 (GraphPad Software, Boston, MA, USA). It should be noted that all receptors were immobilized on the plate surface rather than present in solution; therefore, expressing their coating as a molar concentration does not accurately reflect the number of accessible binding sites. Accordingly, EC₅₀ values were calculated based on the molar concentrations of the soluble ligand applied in the assay.

### Analysis of LRP-mediated uptake of oxAAT using receptor-associated protein (RAP)

We confirmed the specificity of our assay by analyzing LRP1-mediated uptake of oxAAT using specific ligand RAP to block LRP1 binding, as described previously (Cooper et al. 2021). Briefly, macrophages were pretreated with 0.8 µM RAP (Enzo, Long Island, NY, USA) prior to addition of TAMRA-labeled oxAAT and incubated for 2–6 h. Cells were then washed three times with PBS and lysed in RIPA buffer (Thermo Fisher Scientific) supplemented with 1% protease inhibitor cocktail containing inhibitors targeting serine, cysteine, and aspartic proteases (#P8340, Sigma-Aldrich, St. Louis, MO, USA). Lysates were mixed with 4X SDS sample buffer containing β-mercaptoethanol (Thermo Fisher Scientific) and heated at 95 °C for 5 min. Equal amounts of protein (15 µg) were separated on 10% SDS–PAGE gels and transferred onto polyvinylidene difluoride (PVDF) membranes (Merck). TAMRA-oxAAT was detected using a rabbit anti-TAMRA primary antibody (Thermo Fisher Scientific; 1:1000 in TBS-T), followed by a HRP-conjugated anti-rabbit secondary antibody (Cell Signaling, 1:5000 in TBS-T). Images were acquired using a ChemiDoc Touch Imaging System (Bio-Rad, Feldkirchen, Germany).

### Human blood PBMCs and monocyte-derived macrophage cultures

PBMCs were isolated from healthy donors using Lymphosep (CC-Pro, Oberdorla, Germany) according to the manufacturer’s instructions. Different PBMC subtypes were identified based on cell size, morphology, and flow cytometric scatter characteristics (Supplementary figure S1A–C). Cell size distribution, measured with a Cellometer Auto T4 Plus (Nexcelom Bioscience, Lawrence, MA, USA), ranged from ~ 7–15 μm (Supplementary figure S1A), consistent with expected PBMC populations. Cytospin preparations examined by light microscopy (Supplementary figure S1B) allowed morphological identification: smaller cells with dense nuclei and limited cytoplasm corresponded to lymphocytes, whereas larger cells with abundant cytoplasm were monocytes. Flow cytometric analysis of forward scatter (FSC) and side scatter (SSC) further supported these assignments, with lymphocytes showing lower FSC and SSC and monocytes higher FSC and SSC signals (Supplementary figure S1C). Together, these complementary approaches confirmed that the PBMC preparations contained the expected mixture of lymphocytes and monocytes.

For adherent PBMC experiments, 2 × 10⁶ cells were plated on sterile glass coverslips (18 mm diameter) placed in 12-well culture plates. Coverslips were sterilized by autoclaving and briefly flamed before use. All procedures were performed under aseptic conditions in a biosafety cabinet to prevent contamination and avoid unintended cellular activation. Cells were allowed to adhere for 2 h at 37 °C with 5% CO₂. Non-adherent cells were removed, and adherent cells were cultured for 24 h before analysis. For experiments with total PBMCs, 2 × 10⁶ cells were plated on 12 well plates with cell repellent surface (Greiner, Greiner Bio-One, Cellstar^®^, Kremsmünster, Austria). All experiments were performed in RPMI medium without phenol red (Life Technologies) and without serum but supplemented with 1% non-essential amino acids, 2 mM sodium pyruvate, and 20 mM HEPES (Moraga and Janciauskiene 2000).

For monocyte-derived macrophages, PBMCs were seeded in 10 cm dishes at 4 × 10⁷ cells per dish in RPMI-1640 medium containing L-glutamine. After 2 h, non-adherent cells were removed, and the adherent monocytes were cultured in fresh medium supplemented with 5% human AB serum (Pan Biotech, Aidenbach, Germany) and recombinant human macrophage colony-stimulating factor (M-CSF, 25 ng/mL; ImmunoTools, Friesoythe, Germany) for 7 days to induce macrophage differentiation (Pradhan et al. 2024). Differentiated macrophages were washed and detached using accutase enzyme (activity > 500 U/mL), a gentle enzymatic solution in dulbecco’s phosphate-buffered saline without calcium or magnesium, containing 0.5 mM EDTA and phenol red (Pan Biotech). Cells were seeded onto 12-well plates containing 18 mm glass coverslips at 5 × 10⁵ cells per well and were allowed to adhere and recover for 24 h in complete culture medium prior to experiments.

Although antibiotics generally do not alter cell morphology, they may affect cell physiology or interact with other treatments. Typically, reports do not highlight a direct interaction of GR with antibiotics like penicillin and streptomycin. An exception is rifampicin, which can bind and activate human GR (Calleja et al. 1998; Ray et al. 1998). Modern culture conditions are highly sterile, and bacterial contamination is rare, even in long-term cultures. Therefore, to avoid potential confounding, all experiments were performed without antibiotics.

### THP-1 cell culture and differentiation to macrophages

THP-1 cells are a human monocytic leukemia cell line commonly used as a model for monocytes and macrophages. THP-1 cells were cultured in RPMI-1640 medium with L-glutamine (Thermo Fisher Scientific) supplemented with 10% fetal bovine serum (FBS, Pan Biotech) at 37 °C and 5% CO_2_. THP-1 cells were differentiated into macrophages by treatment with phorbol 12-myristate-13-acetate (PMA, 100 ng/mL; Sigma-Aldrich) for 48 h, followed by a 24 h resting period in PMA-free medium prior to experimentation (Wang et al. 2023). For microscopy, cells were seeded onto 18 mm glass coverslips in 12-well plates (2 × 10⁵ cells/well). For co-immunoprecipitation experiments, cells were seeded in 6-well plates (5 × 10⁵ cells/well).

### Co-immunoprecipitation of GR and AAT in THP-1 cell-derived macrophages

Co-immunoprecipitation was performed based on Bai et al. (Bai et al. 2022). THP-1-derived macrophages were lysed in 200 µL ice-cold lysis buffer per well (6-well plate) containing 50 mM Tris-HCl (pH 7.4), 0.5% IGEPAL CA-630 (2-[4-(2,4,4-trimethylpentan-2-yl)phenoxy]ethan-1-ol; a non-ionic, non-denaturing detergent), 150 mM NaCl, 1 mM EDTA, 2 mM sodium orthovanadate, and 1% protease inhibitor cocktail. Lysates were incubated on ice for 30 min with vortexing every 10 min and clarified by centrifugation at 17,000 × g for 15 min at 4 °C. Supernatants were collected, and protein concentrations were determined using the BCA assay.

For immunoprecipitation, 2 µg of rabbit anti-AAT (Thermo Fisher Scientific) or rabbit anti-GR antibody (Thermo Fisher Scientific) was added to equal amounts of lysate protein and incubated overnight at 4 °C with gentle rotation. Rabbit IgG (Abcam) served as isotype control. Protein A sepharose beads (Cytiva) were washed and 20 µl of beads were added to each sample, followed by incubation for 2 h at 4 °C. Beads were washed four times with Tris buffer (10 mM, pH 7.4) containing protease inhibitors. Bound proteins were eluted in 2× SDS sample buffer with β-mercaptoethanol and heated for 5 min at 95 °C. Samples were separated by 7.5% SDS-PAGE, transferred to PVDF membranes, and analyzed by immunoblotting using anti-GR (Cell Signaling) or anti-AAT (Thermo Fisher Scientific) antibodies. Bands were detected using HRP-conjugated secondary Veriblot antibody (Abcam) and enhanced chemiluminescence and imaged with a ChemiDoc system.

### Immunofluorescence co-staining of AAT and GR

Adherent cells were fixed with 4% paraformaldehyde (PFA) for 10 min at 37 °C, permeabilized with 0.05% Triton-X100 for 5 min and blocked with 5% BSA in PBS for 1 h. AAT was stained with monoclonal mouse-anti AAT antibody (Proteintech, dilution 1:200) and GR was stained with polyclonal rabbit anti-GR antibody (Thermo Fisher Scientific, dilution 1:100) or monoclonal rabbit anti-GR antibody (Cell Signaling, dilution 1:200). For visualization, cover slips were incubated with secondary Alexa Fluor-488 donkey anti-rabbit and Alexa Fluor-594 donkey anti-mouse antibodies for 1 h at room temperature and nuclei were stained with 4`,6-Diamidino-2-phenylindol (DAPI, Thermo Fisher Scientific). The coverslips were mounted using fluoromount W mounting medium and images were acquired using the FluorView 1000 (Olympus, Tokio, Japan) confocal laser scanning microscope equipped with a 60x oil immersion objective.

### Analysis of *NR3C1* (GR) gene expression in human PBMCs

Total PBMCs were left untreated or were treated with nAAT (1 mg/mL), oxAAT (1 mg/mL) or dexamethasone (Dex, 100 nM, Sigma-Aldrich) for 24 h at 37 °C, 5% CO₂. For gene expression analysis, total RNA was isolated using the RNeasy Mini Kit (Qiagen, Venlo, The Netherlands) according to the manufacturer’s instructions. RNA was reverse transcribed using the high-capacity cDNA reverse transcription kit (Applied Biosystems, Thermo Fisher Scientific, Waltham, MA, USA) and cDNA was amplified using Taqman gene expression assays (*NR3C1*: Hs00353740_m1, *HPRT*: Hs02800695_m1, Applied Biosystems) on a StepOnePlus Real-Time PCR Systems machine (Applied Biosystems). Expression of *NR3C1* was calculated relative to the house-keeping gene *HPRT* with the 2^(−∆Ct)^ method (∆Ct = Ct value target gene − Ct value of house-keeping gene). All measurements were performed in duplicates from 2 to 3 independent experiments.

### Statistical analysis

All data are presented as means ± SD or SEM, as stated in the figure legends. Statistical analyses were performed using GraphPad Prism (Version 10.6.1 (892)). Methods of statistical analyses were chosen based on the design of each experiment as detailed in the figure legends. If not stated otherwise, *p* < 0.05 was considered statistically significant.

## Results and discussion

GR is a ligand-activated transcription factor that controls both pro- and anti-inflammatory gene networks to maintain immune homeostasis (Plumb et al. 2013). A recent study showed that AAT interacts with GR (Bai et al. 2022) and proposed a link between AAT and GR-dependent regulation of inflammation (Bai et al. 2024). The AAT–GR axis is of significant scientific and clinical interest, as it links two endogenous anti-inflammatory systems: the serine protease inhibitor network and glucocorticoid signaling. This functional interplay prompted us to perform independent experiments to validate and extend these findings. First, we show that not only nAAT but also oxAAT binds GR. Second, by comparing native, oxidized, and cleaved AAT, we found that GR binding requires structural integrity, as cleaved AAT did not bind. Third, we evaluated GR binding in comparison with other candidate AAT receptors (LRP1, SR-B1, TfR, and CD36), placing GR within a broader receptor context. Finally, cytosolic AAT-GR colocalization was observed in non-activated human PBMCs but not in vitro-differentiated macrophages, where GR was predominantly nuclear. In addition, AAT, like dexamethasone, reduced *NR3C1* mRNA expression in PBMCs.

An interaction between AAT and GR in vitro was shown to occur with submicromolar affinity (EC₅₀ ≈ 50 nM) as determined by microscale thermophoresis (MST) (Bai et al. 2022). MST allows direct, immobilization-free measurements, minimizing artifacts that may arise from protein immobilization. We have previously applied MST to study serpin-glycosaminoglycan interactions (Ulbricht et al. 2017); however, in this study, we employed an ELISA-based binding assay to enable parallel comparison of AAT binding to GR and other candidate receptors (Fig. 1). Moreover, we examined both nAAT and oxAAT, as oxidation occurs in vivo and has been detected in therapeutic AAT preparations (Magallón et al. 2021; Rahman and Adcock 2006). To confirm the reliability of our assay, we evaluated the interaction between the LRP1 and the AAT-elastase complex (serpin–elastase complex, SEC), a well-established positive control (Table 1). LRP1 (also known as CD91) is a signaling receptor, modulating intracellular pathways involved in inflammation, tissue remodeling, and cell survival (Strickland et al. 2002; Zou et al. 2026) and it is a scavenger receptor (Yamamoto et al. 2024) mediating endocytosis and clearance of SECs (Strickland et al. 2011; Herz and Strickland 2001). Mechanistically, LRP1 binds with high affinity to covalently stabilized SECs formed when a serpin inhibits its target protease, whereas native serpins alone interact only weakly (Jensen et al. 2009). This selectivity enables efficient clearance of SECs while preserving functional inhibitors. For our experiments we used LRP1-C4, which corresponds to the recombinant cluster IV of the ligand-binding region of LRP1. The extracellular domain of LRP1 contains ~ 40 cysteine-rich complement-type repeats organized into four clusters (I–IV), with clusters II–IV forming the main ligand-binding regions. Cluster IV, located closest to the cell membrane, mediates interactions with several ligands (Bres and Faissner 2019). Recombinant LRP1-C4 includes the complement-like repeats responsible for ligand binding but lacks additional domains, such as the epidermal growth factor-like repeats. LRP1-C4 was therefore used in our ELISA-based assay as a simplified model to specifically assess ligand interactions.

As expected, the AAT-elastase complex exhibited high-affinity binding to LRP1 (EC₅₀ = 0.17 µM) whereas nAAT and oxAAT bound LRP1 with substantially lower affinity (2.9 µM and 1.3 µM, respectively; Fig. 1C, Table 1). To exclude potential interference by the anti-AAT antibody, we repeated the assays using TAMRA-labeled AAT together with an anti-TRITC antibody specific to the fluorophore. This approach was previously used to quantify vaspin (SERPINA12)-LRP1 binding (Tindall et al. 2024). As shown in Fig. 1C, the AAT-elastase (SEC)-LRP1 interaction closely matched the antibody-based measurements and exhibited an EC₅₀ of approximately 0.23 µM, consistent with the values in Table 1.

**Table 1:** Overview of EC_50_ values for all AAT preparations and receptors tested in this study

	nAAT	oxAAT	SEC
LRP1	2.9 (2.5–3.3)	1.3 (1.1–1.4)	0.17 (0.15–0.19)
GR	0.9 (0.7–1.2)	2.6 (2.3–2.9)	no interaction
SR-BI	> 5	2.6 (2.0–3.3)	not tested
TfR	> 5	> 6	not tested
CD36	> 7	> 5	not tested

All EC_50_ (µM) data are presented as with 95% confidence interval, derived from non-linear regression of binding curves obtained from at least two independent experiments. n/oxAAT – native/oxidized AAT; SEC – serpin-elastase complex; LRP1 - low-density lipoprotein receptor-related protein 1; GR: glucocorticoid receptor; SR-B1: scavenger receptor class B type 1; TfR: transferrin receptor; CD36 - cluster of differentiation 36

**Fig. 2:**
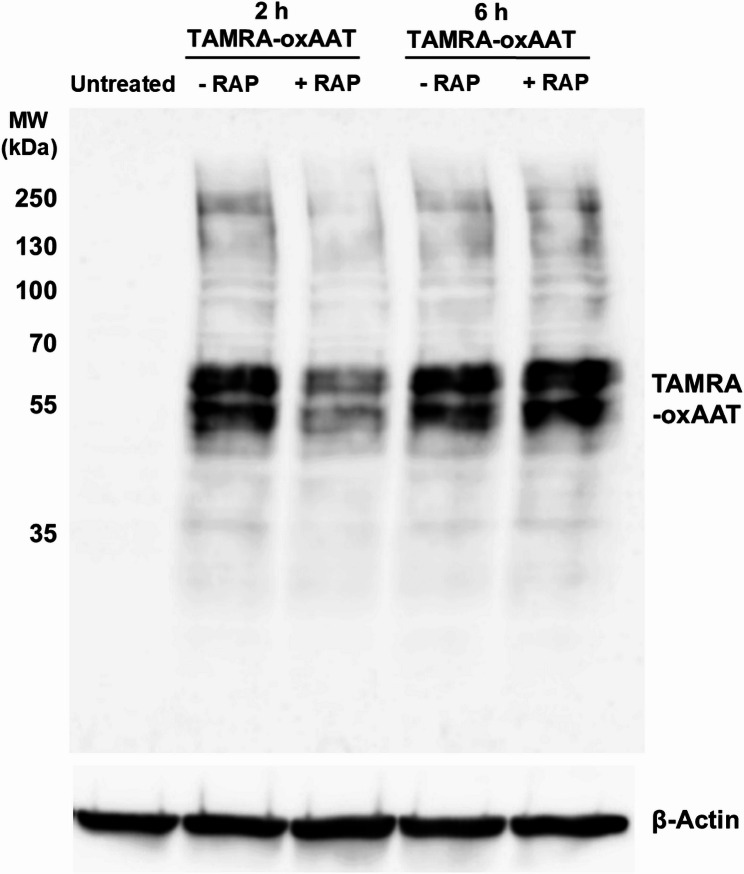
RAP reduced cell-associated oxAAT in THP-1 macrophage lysates. RAP (receptor-associated protein) was used as a competitive binding protein to confirm LRP1-specific interactions. THP1-derived macrophages were left untreated or were pre-treated with the LRP1-blocker RAP (0.8 µM) for 30 min, prior to addition of 0.1 mg/mL TAMRA-labelled oxAAT for 2–6 h. Following, cells were washed, lysed and equal amounts of lysates (15 µg) were separated on 10% SDS-PAGE. Cellular TAMRA-oxAAT was visualized using a rabbit anti-TAMRA primary antibody and a HRP-coupled anti-rabbit secondary antibody. β-actin served as a loading control

We finally validated our assay by confirming that LRP1 mediates oxAAT cellular association. Pretreatment of macrophages with the LRP1 ligand RAP (Prasad et al. 2015) markedly reduced TAMRA-labeled oxAAT uptake at 2 h (Fig. 2). In contrast, the residual uptake observed at 6 h likely reflects low-affinity binding and/or engagement of alternative, LRP1-independent pathways, consistent with previous reports (Cooper et al. 2021; Lillis et al. 2008).

Using this validated experimental setup, we found that both, nAAT and oxAAT, bind the GR, with EC₅₀ values of 0.9 µM and 2.6 µM, respectively (Fig. 1D; Table 1). Although both forms interact with GR, the roughly threefold difference in EC₅₀ values indicates that oxidative modification may influence this interaction. Oxidation of AAT primarily affects Met residues within the reactive center loop but also other exposed regions of the protein, potentially inducing conformational changes that might partially alter receptor recognition and binding affinity. These differences between nAAT and oxAAT should also be interpreted in the context of physiological AAT concentrations. Native AAT circulates at relatively high plasma levels (~ 19–38 µM), whereas oxAAT typically represents 5–10% of total AAT under basal conditions but can rise substantially during oxidative stress and inflammation (Bergin et al. 2012). Therefore, the circulating levels of native and oxAAT are likely sufficient to mediate GR interactions under normal and stress conditions.

Consistent with previous reports (Lockett et al. 2015; Graziadei et al. 1998; Siebers et al. 2018), weak interactions (EC₅₀ >5 µM) of nAAT and oxAAT were also observed with SR-B1, TfR, and CD36 receptor (Fig. 1E–G; Table 1). For these receptor interactions, binding curves of nAAT and oxAAT did not reach a clear plateau within the tested concentration range and EC₅₀ values rather represent estimates.

In contrast, elastase-cleaved oxAAT fragments and the synthetic C36 peptide of AAT did not bind GR, as evidenced by the absence of a detectable GR binding signal (Fig. 1D; Supplementary figure S2). Similarly, AAT complexed with elastase (SEC), which involves AAT cleavage during complex formation, showed no binding to GR (data not shown). These findings indicate that AAT-GR interaction is independent of AAT protease-inhibitory activity but requires structural integrity of the protein. It is important to note that no additional protease inhibitors were added to the AAT-elastase mixtures, and residual elastase activity was not specifically quantified. Thus, although interference from residual elastase cannot be formally excluded, it is considered unlikely to have influenced the observed results. Specifically, AAT-elastase complexes (containing cleaved AAT and residual non-inhibited elastase) showed the expected binding to LRP1, but not to GR, whereas preparations containing cleaved AAT with a fraction of non-inhibited elastase did not show GR interaction. Moreover, if significant residual elastase activity had caused nonspecific proteolysis of assay components (e.g., immobilized proteins in ELISA wells), broader changes in binding signals would be expected, especially at high concentrations of the AAT-SEC tested for LRP1 binding; however, this was not a case. In support, historical publication by John R. Hubbard I and Mohammed Kalimi reported that GR is relatively resistant to degradation by serine proteases (Hubbard and Kalimi 1985).

Human macrophages and other immune cells express the *SERPINA1* gene and produce AAT, albeit at substantially lower levels than hepatocytes (Stockley 2015; Janciauskiene et al. 2011). Locally synthesized AAT may remain intracellular or be secreted into the surrounding microenvironment, where it can contribute to autocrine or paracrine regulation of protease activity and inflammation. In immune cells, GR predominantly resides in the cytoplasm in complex with chaperones and co-chaperones (Faught and Schaaf 2023; Desgeorges et al. 2019; Pratt and Toft 2003), which maintain the receptor in a ligand-accessible conformation. Thus, endogenously produced AAT may have access to cytoplasmic GR. Consistent with this hypothesis, Bai et al. reported an interaction between macrophage-derived AAT and GR (Bai et al. 2022).

**Fig. 3:**
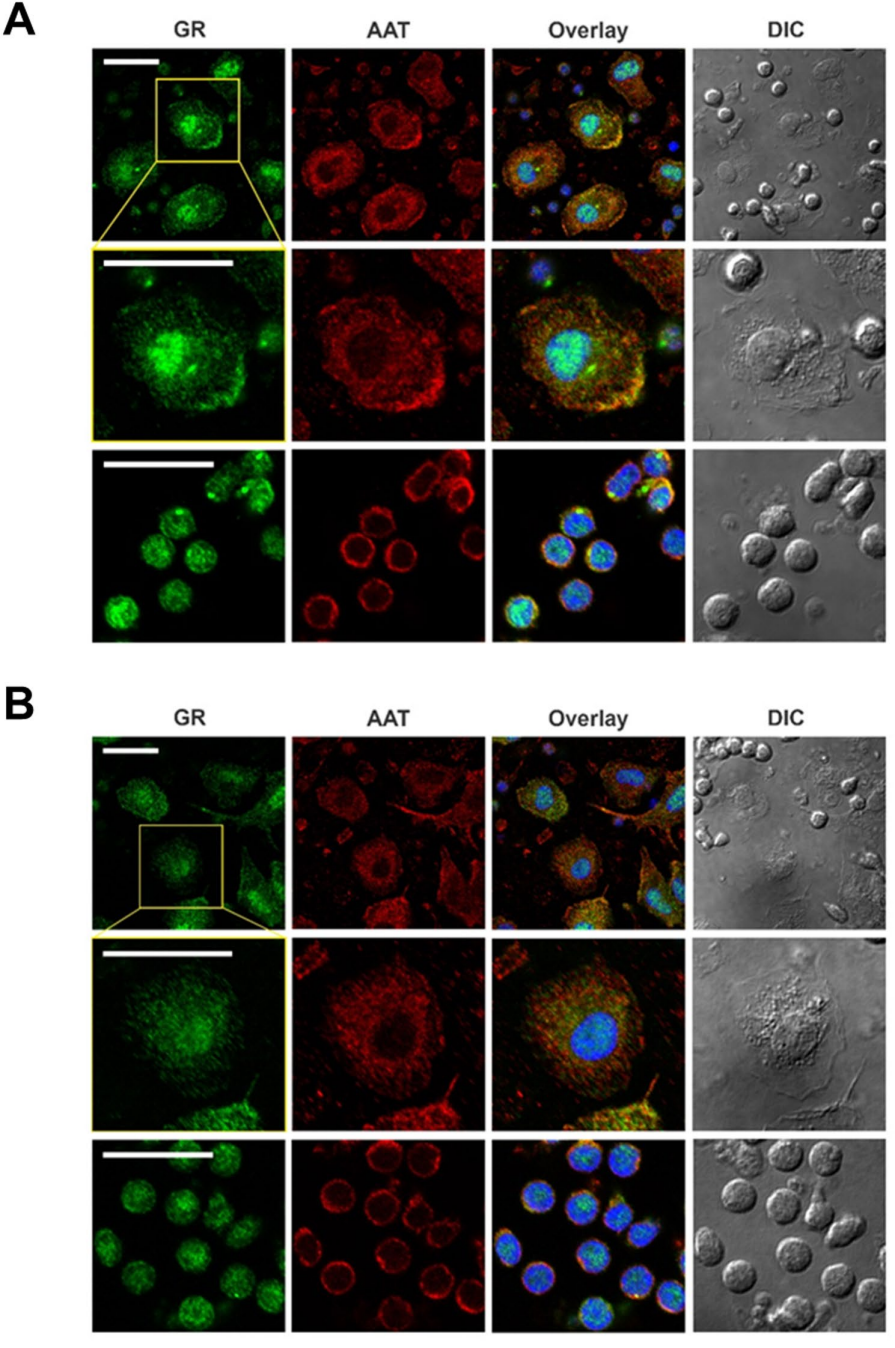
Endogenous AAT and GR co-localize in human PBMCs. Co-staining of adherent human blood PBMCs with anti-AAT (*red*) and polyclonal (**A**) or monoclonal (**B**) anti-GR (*green*) antibodies reveals co-localization of AAT and GR in the cytoplasm. Nuclei were stained with DAPI. Images were taken using a confocal laser scanning microscope (Olympus FluorView 1000) equipped with a 60× oil-immersion objective. Scale bars represent 20 μm

We used adherent human PBMCs, as GR is expressed across the major subtypes of PBMCs, including T cells (both CD4 + and CD8+), B cells, dendritic cells and monocytes (Nicolaides et al. 2010; Miller et al. 1998) (Supplementary figure S1). PBMCs were cultured without antibiotics using phenol red-free and serum-free medium, to prevent spontaneous GR activation, ensuring that GR remains predominantly cytoplasmic (Raivio et al. 2002; Lee et al. 2022). Under these conditions, AAT and GR showed clear cytosolic colocalization in multiple PBMC subtypes, supporting a GR-AAT intracellular interaction (Fig. 3). It is important to note that these studies were limited to total endogenous AAT, without distinguishing between oxidized and native forms, as specific antibodies capable of differentiating oxAAT from nAAT are currently unavailable.

We also found that both nAAT and oxAAT reduce *NR3C1* mRNA levels (~ 40%), within 24 h of cell culture (Supplementary figure S3). Under the same experimental conditions, dexamethasone used as a typical ligand for GR, also reduced *NR3C1* mRNA expression (~ 50%, *p* < 0.001, Supplementary figure S3). This latter is consistent with previous reports showing that dexamethasone can down-regulate GR expression in PBMCs after short-term exposure, reflecting ligand-dependent feedback regulation of GR signaling (Lockett et al. 2024; Bileck et al. 2014).

**Fig. 4:**
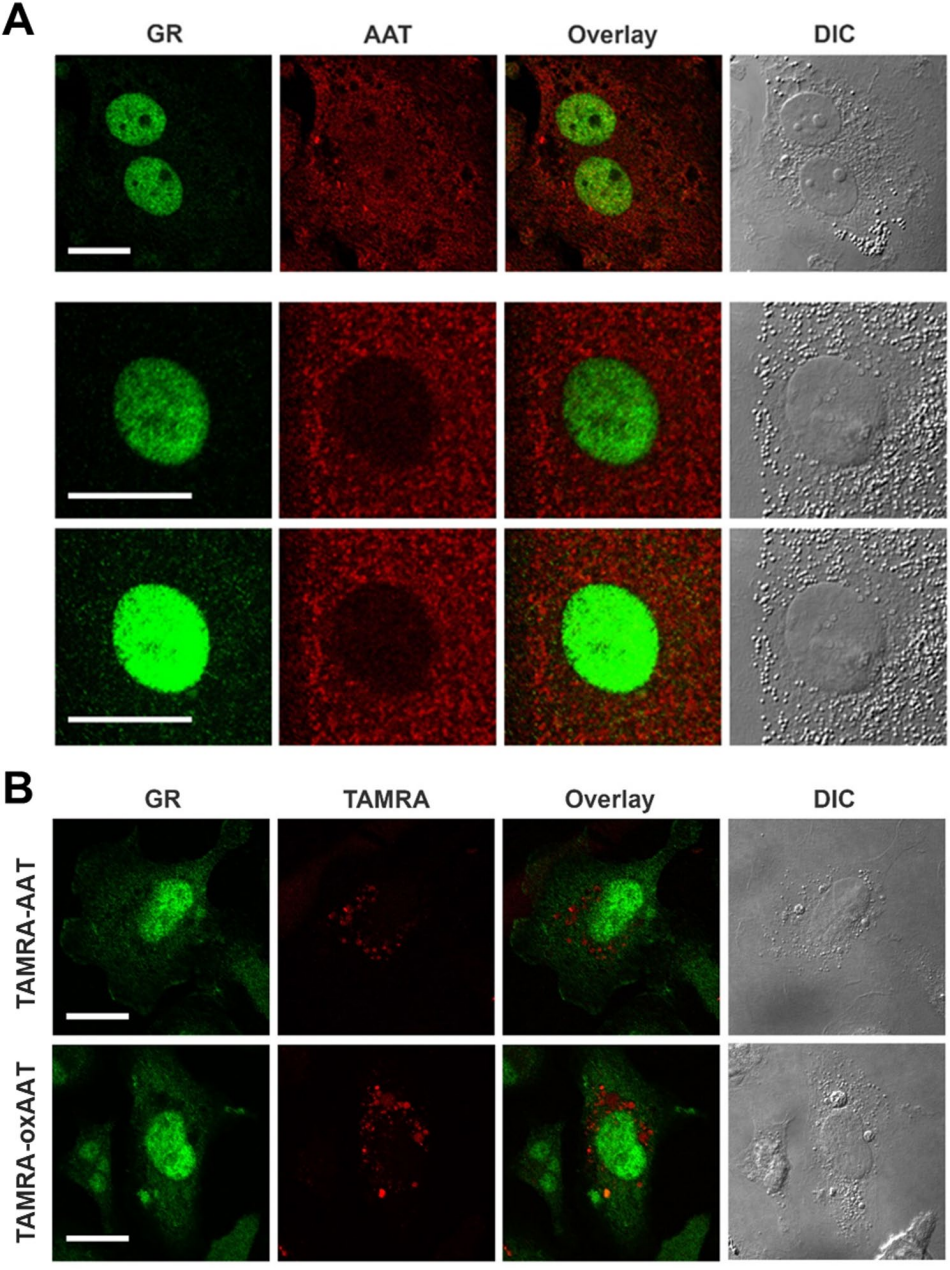
No colocalization between endogenous and exogenous AAT and GR in THP-1 macrophages. **A** Co-staining with anti-AAT (*red*) and anti-GR (*green*) antibodies showed no co-localization between endogenous AAT and GR in THP-1derived macrophages. **B** THP-1 derived macrophages were treated with 0.1 mg/mL TAMRA-labelled nAAT or oxAAT for 18 h and GR was visualized using an anti-GR antibody which also showed no co-localization between exogenous AAT/oxAAT and GR. Images were acquired using a confocal laser scanning microscope (Olympus FluorView 1000) equipped with a 60× oil-immersion objective. Scale bars represent 20 μm

**Fig. 5:**
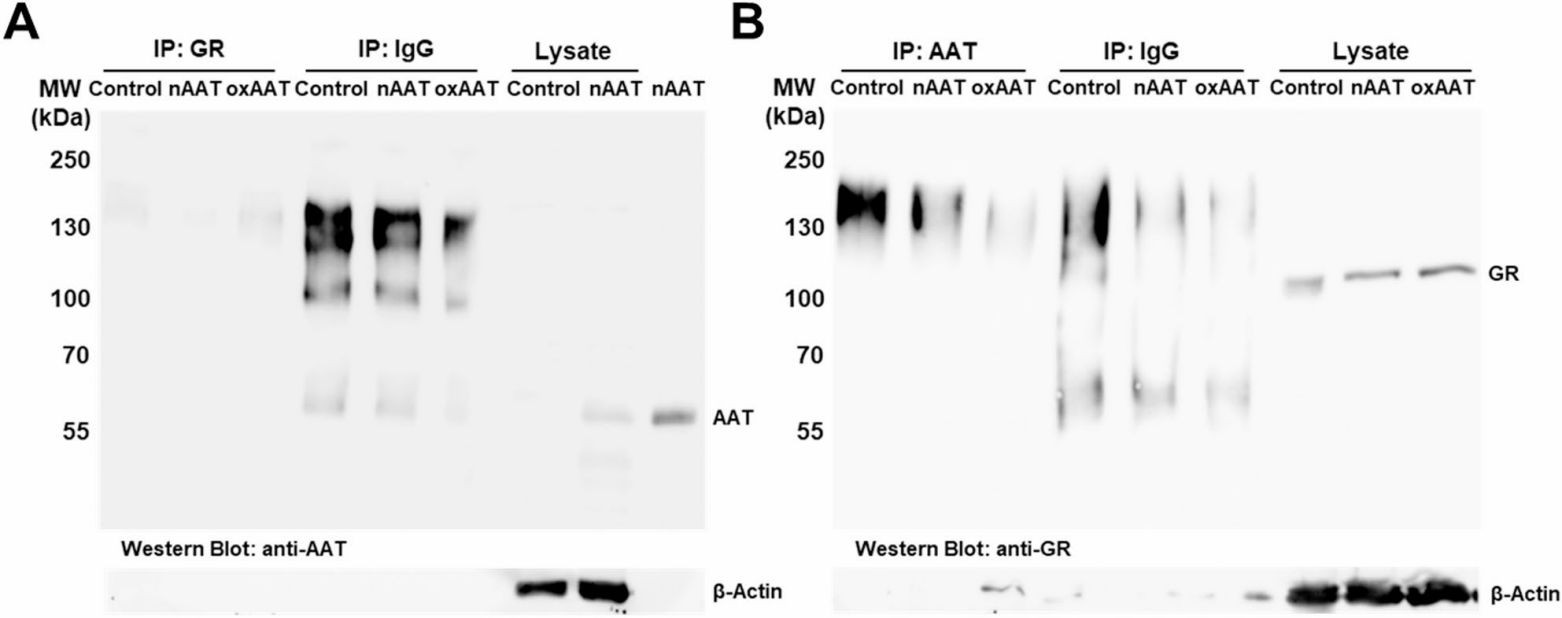
Co-immunoprecipitation (Co-IP) analysis showed no detectable complexes between nAAT or oxAAT and GR in THP-1–derived macrophages. Cells were left untreated (control) or treated with 0.5 mg/mL nAAT or oxAAT for 4 h. Cell lysates were prepared, Co-IP was performed, and samples were separated on 7.5% SDS–PAGE gels. **A** Immunoprecipitation with an anti-GR antibody followed by Western blotting with an anti-AAT antibody. **B** Immunoprecipitation with an anti-AAT antibody followed by Western blotting with an anti-GR antibody. Non-immune IgG was used as a negative control for immunoprecipitation. 20 µL of immunoprecipitated samples were loaded per lane, together with 12 µg of total cell lysate (**A** and **B**) and 100 ng of nAAT (**A**). Blots were reprobed with anti-β-actin. One representative experiment out of three independent experiments is shown

We next examined whether AAT colocalizes with GR in THP-1- and human monocyte-derived macrophages. THP-1-derived macrophages were additionally treated with TAMRA-labeled nAAT or oxAAT (0.1 mg/mL, 18 h) and stained for GRα/GRβ, followed by confocal microscopy. In all conditions, GR was predominantly nuclear, whereas AAT was cytoplasmic, and no AAT-GR colocalization was observed. (Fig. 4A–B). Co-immunoprecipitation in THP-1-derived macrophages treated with nAAT or oxAAT (0.5 mg/mL, 4 h) confirmed the absence of AAT-GR interaction (Fig. 5A–B). Similarly, no colocalization was observed in human monocyte-derived macrophages (Supplementary figure S4). The lack of colocalization or interaction between GR and either endogenous or exogenous AAT in our macrophage models may be attributed to several factors. First, PMA-induced differentiation of THP-1 monocytes into macrophages activates GR through PKC-dependent signaling, leading to its nuclear localization and promoting a partially activated, pro-inflammatory phenotype (Castrillo et al. 2001). Second, macrophages cannot be maintained under completely serum-free conditions; thus, macrophage growth factors in the culture media and endogenous glucocorticoids in the serum may further promote GR nuclear accumulation (Wang et al. 2026). Third, unlike previous macrophage models used to demonstrate AAT-GR interactions, our experiments were performed without antibiotics. As antibiotics can alter cellular metabolism, stress responses, and activation, (Cifarelli et al. 1982; Yang et al. 2017) their absence may influence AAT-GR interaction. Collectively, these factors suggest that macrophage-specific signaling, growth factor exposure, and culture conditions can obscure AAT-GR interactions, highlighting the importance of cell type and experimental context in studying this pathway.

In summary, our results demonstrate that both native and oxidized AAT interact with GR in vitro and in cellular systems, with binding restricted to the intact protein. A limitation of this study is that we focused primarily on AAT-GR interaction and colocalization without investigating downstream signaling, although we show that AAT, similar to dexamethasone, reduces *NR3C1* (GR) mRNA levels. Moreover, all experiments were performed under controlled in vitro conditions, which may not fully capture the complexity of in vivo environments. Nonetheless, these findings identify the AAT-GR axis as a point of convergence between protease inhibition and transcriptional regulation of inflammation. Further elucidation of this crosstalk may reveal novel mechanisms of endogenous immune regulation and contribute to understanding inter-individual variability in glucocorticoid responsiveness.

## Supplementary Information

Supplementary Material 1.

## Data Availability

All data generated or analyzed during this study are included in this published article and its supplementary information files.

## Abbreviations

AAT: Alpha1-antitrypsin
ADAM-17: A disintegrin and metalloprotease 17
CD: Cluster of differentiation
CI: Confidence interval
COPD: Chronic obstructive pulmonary disease
DAPI: 4`,6-Diamidino-2-phenylindol
EC50: Half maximal effective concentration
ELISA: Enzyme-linked Immunosorbent Assay
GR: Glucocorticoid receptor
HSP: Heat shock protein
IL: Interleukin
LRP1-C4: Low-density lipoprotein receptor-related protein 1 cluster IV
M-CSF: Macrophage colony-stimulating factor
Met: Methionine
NF-κB: nuclear factor kappa-light-chain-enhancer of activated B cells
n: native
NCS: N-chlorosuccinimide
NR3C1: Nuclear receptor subfamily 3, group C, member 1
ox: oxidized
PBS: Phosphate-buffered saline
E: porcine pancreas elastase
RAP: Receptor-associated protein
SEM: Standard error of the mean
SERPIN: Serine protease inhibitor
SEC: Serpin-elastase complex
SR-B1: Scavenger receptor class B type 1
TACE: Tumor necrosis factor-α-converting enzyme
TAMRA: Tetramethylrhodamine-5(6)-C2-maleimide
TfR: Transferrin receptor
TNFA: Tumor necrosis factor-α
TRITC: Tetramethylrhodamine
