# Supplementary material for: Alpha-1 antitrypsin–glucocorticoid receptor axis: a new pathway in immune modulation
Source: Mol Med. 2026 Apr 1;32:55. doi: 10.1186/s10020-026-01470-z (PMC13064270; doi:10.1186/s10020-026-01470-z)
Supplement: Supplementary file 1 — Supplementary Material 1. [file 10020_2026_1470_MOESM1_ESM.docx]

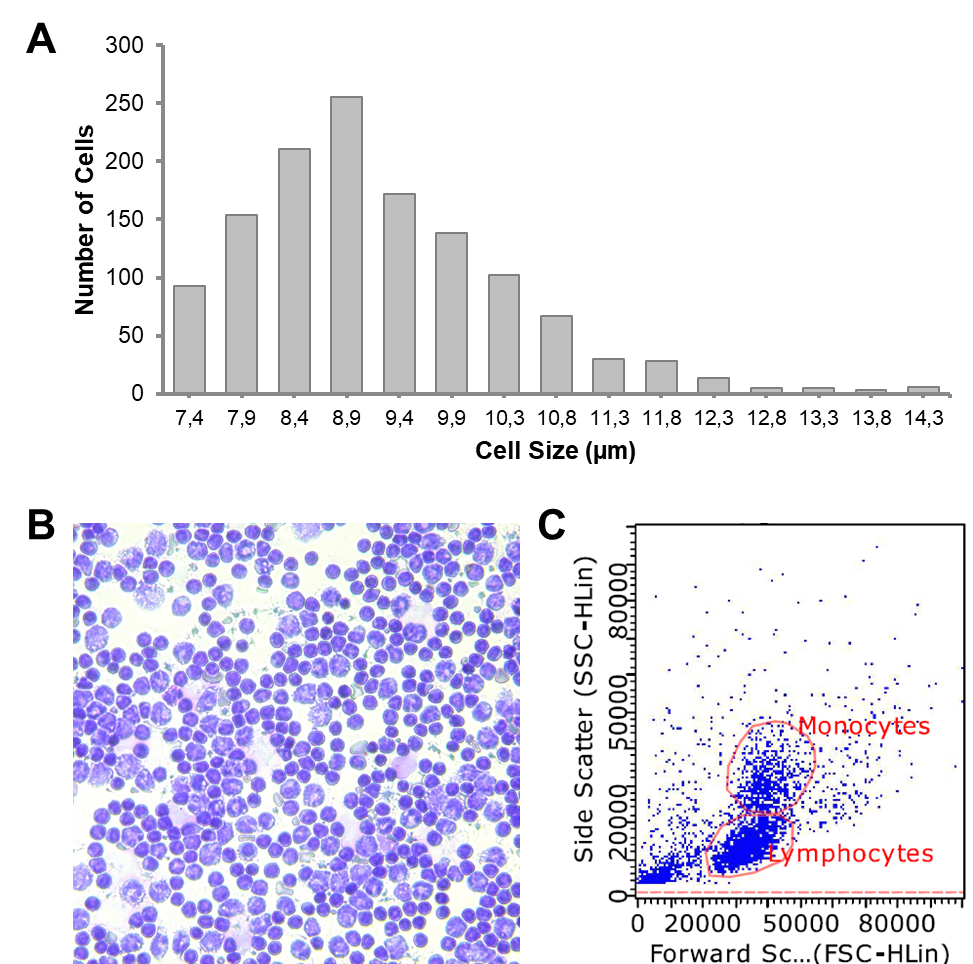


Supplementary figure S1: **Characterization of PBMCs.** (**A**) Cell size chart from Cellometer (Nexolom Lawrence, MA, USA). (**B**) Cytospin stained with May Grünwald and Giemsa. (**C**) Distribution of PBMCs in side and forward scatter by flow cytometry (Guava EasyCyte, Cytek Biosciences, Fremont, CA, USA).


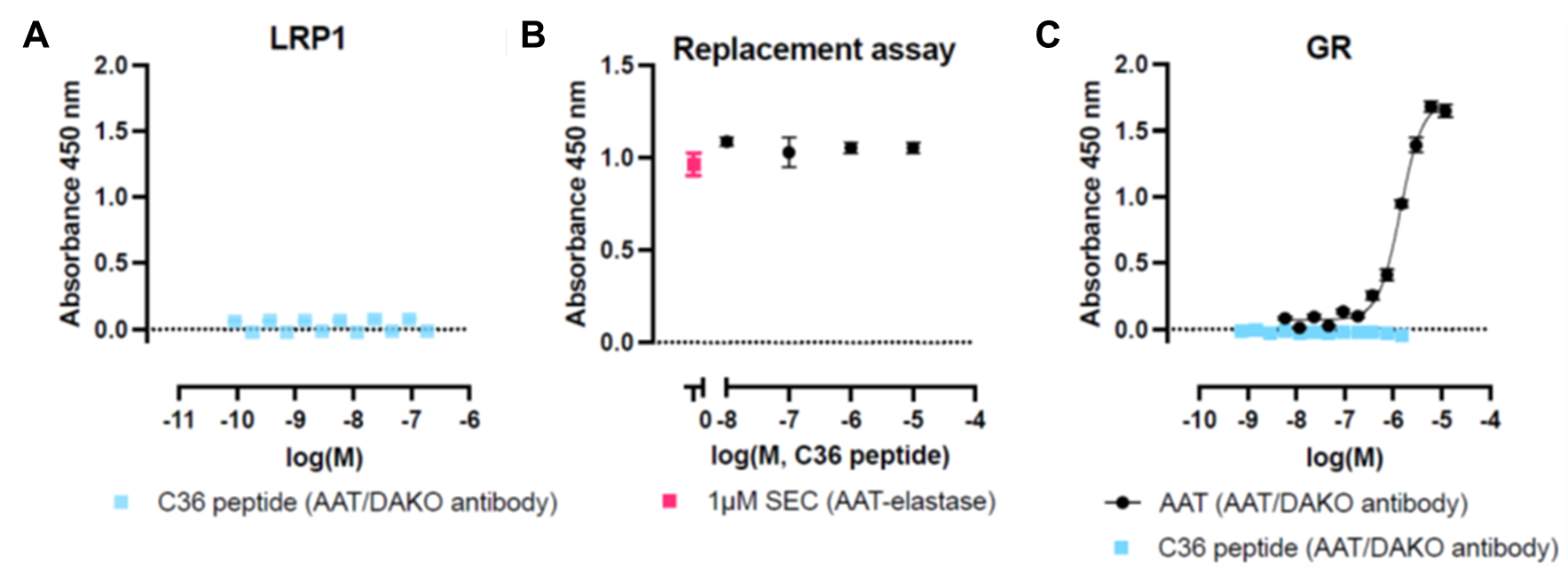


Supplementary figure S2: **ELISA-based binding studies of AAT peptide C36.** (**A**) C36 shows no binding to LRP1-C4 (cluster IV). (**B**) Increasing concentrations of C36 do not replace or suppress SEC binding to LRP1. (**C**) AAT binds GR, but not AAT-derived peptide C36. Data for C36 are presented as mean ± SEM from one experiment, with a minimum of four technical replicates.

**
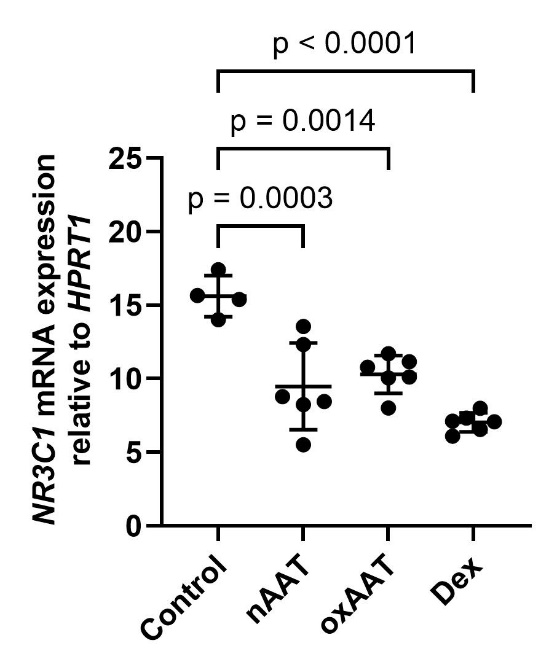
**

Supplementary figure S3: ***NR3C1* mRNA expression is reduced in PBMCs upon nAAT and oxAAT treatment.** PBMCs were left untreated or were treated with nAAT (1 mg/mL), oxAAT (1 mg/mL) or Dex (100 nM) for 24 h at 37 °C/5% CO_2_. *NR3C1* gene expression was analyzed relative to the house keeping gene *HPRT1* by quantitative real-time PCR. Normally distributed data are presented as mean ± SD with 2 repeats from 2-3 independent experiments and statistical significance was analyzed by a One-way ANOVA followed by Tukey multi-comparison test. A p-value < 0.05 was considered significant.


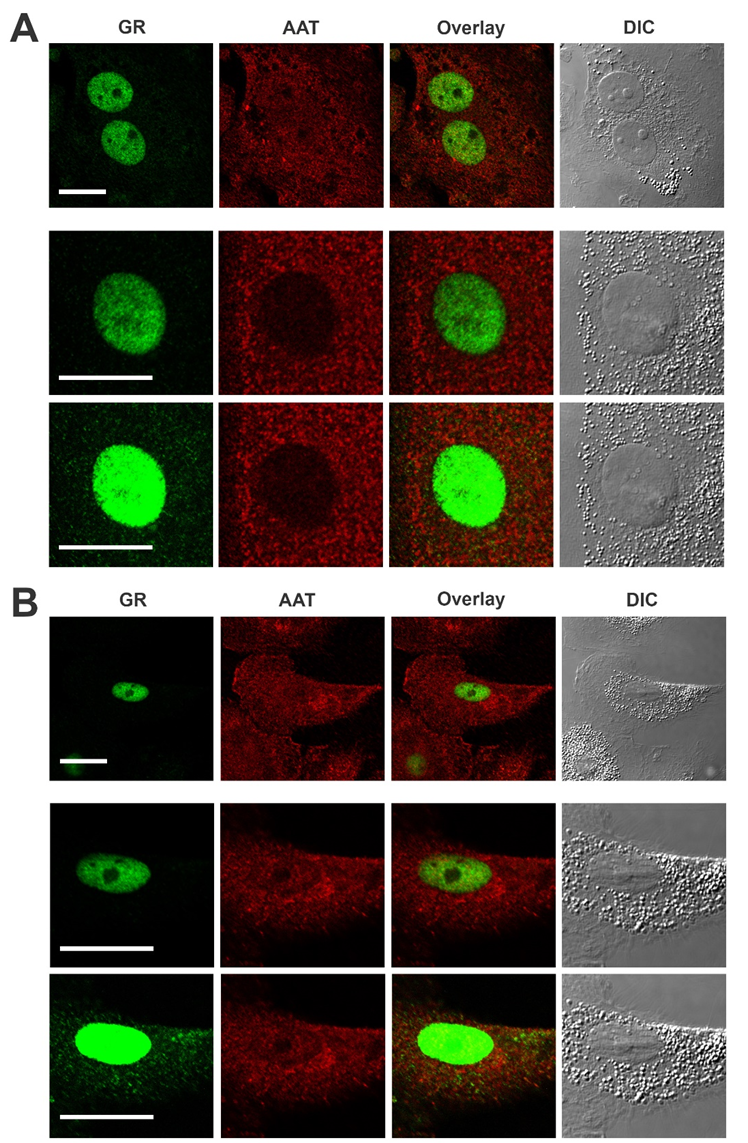


Supplementary figure S4: **No colocalization between endogenous AAT and GR in human blood monocyte-derived macrophages**. Co-staining with anti-AAT (*red*) and anti-GR (*green*) antibodies showed no co-localization between endogenous AAT and GR in human blood monocyte-derived macrophages. Images were acquired using a confocal laser scanning microscope (Olympus FluorView 1000) equipped with a 60× oil-immersion objective. Scale bars represent 20 µm.
